# Failure Mode and Effects Analysis on the Air System of an Aero Turbofan Engine Using the Gaussian Model and Evidence Theory

**DOI:** 10.3390/e25050757

**Published:** 2023-05-06

**Authors:** Yongchuan Tang, Yonghao Zhou, Ying Zhou, Yubo Huang, Deyun Zhou

**Affiliations:** 1 School of Microelectronics, Northwestern Polytechnical University, Xi’an 710072, China; 2 School of Computer Science, Northwestern Polytechnical University, Xi’an 710072, China; 3 School of Electronics and Information, Northwestern Polytechnical University, Xi’an 710072, China; 4 School of Engineering, University of Warwick, Coventry CV4 7AL, UK

**Keywords:** Dempster–Shafer evidence theory, failure mode and effects analysis, Gaussian model, risk management, aero turbofan engine

## Abstract

Failure mode and effects analysis (FMEA) is a proactive risk management approach. Risk management under uncertainty with the FMEA method has attracted a lot of attention. The Dempster–Shafer (D-S) evidence theory is a popular approximate reasoning theory for addressing uncertain information and it can be adopted in FMEA for uncertain information processing because of its flexibility and superiority in coping with uncertain and subjective assessments. The assessments coming from FMEA experts may include highly conflicting evidence for information fusion in the framework of D-S evidence theory. Therefore, in this paper, we propose an improved FMEA method based on the Gaussian model and D-S evidence theory to handle the subjective assessments of FMEA experts and apply it to deal with FMEA in the air system of an aero turbofan engine. First, we define three kinds of generalized scaling by Gaussian distribution characteristics to deal with potential highly conflicting evidence in the assessments. Then, we fuse expert assessments with the Dempster combination rule. Finally, we obtain the risk priority number to rank the risk level of the FMEA items. The experimental results show that the method is effective and reasonable in dealing with risk analysis in the air system of an aero turbofan engine.

## 1. Introduction

Risk analysis and management under uncertainty is an important issue in many practical applications such as aircraft components [1,2], motors [3], and so on [4]. Failure mode and effects analysis (FMEA) is an useful method to predict risks and make preventions in advance. It obtains the risk priority number (RPN) value of each FMEA item and then uses the RPN to rank and manage potentially uncertain risk items [5,6]. FMEA is widely used in various fields to deal with risk prediction and management [7,8]. For example, Zhang et al. [9] combined the FMEA method and fault tree analysis (FTA) to analyse the ignition source fail-safe feature and the improved method was applied to an aircraft fuel tank system with effective experimental results. Ahn et al. [10] used the fuzzy-based FMEA model for a hybrid molten carbonate fuel cell and gas turbine system for marine propulsion. Liu et al. [11] presented an improved FMEA method for a shipboard integrated electric propulsion system, combining both the fuzzy logical method and DEMATEL theory. In [12], a new integrated fuzzy smart FMEA framework, combining the fuzzy set theory, analytical hierarchy process (AHP), and data envelopment analysis (DEA) was proposed in a processing risk analysis of an aircraft landing systems, where these three algorithms were used to handle uncertainty and enhance the reliability of the risk assessment. The data-driven FMEA method was proposed for maintenance planning in the aviation industry [13]. Qin et al. [14] applied the FMEA method for risk evaluation of a steam valve system by combining interval type-2 fuzzy sets with an evidential reasoning approach. Nicolin et al. [15] used the FMEA method for a military nose landing gear project to enhances the quality, reliability and safety of the project. FMEA has also been successfully deployed to determine the risk that causes failures of the pneumatic systems of a computer numerical control milling machine [16] and analyse the risk of the electronic circuit by calculating the severity, probability and detection rating [17]. In this work, we apply an improved FMEA method to the air system of an aero turbofan engine.

Many works have applied considerable methods to improve the FMEA theory in different systems’ risk analysis and management for more effective risk analysis and management [18,19]. Thus, many hybrid FMEA methods have been proposed that combine the advantages of different theories and methods. Yazdi et al. [20] proposed a conventional and fuzzy-based FMEA method sensitive to each input including language variables and the weigh of specialists. The new method effectively addressed some flaws of the classical FMEA model. Fan et al. [21] proposed an improved risk priority number model of FMEA by using best–worst approaches based on D numbers. In [22], a new FMEA model integrating linguistic Z-numbers and projection was proposed, which proved to be practical and flexible when used in an aircraft landing system. Bhattacharjee et al. [23] improved the FMEA model by using interval number-based logistic regression theory. Li et al. [24] proposed an AHP-FMEA to analyse the failure reasons of floating offshore wind turbines including main components, critical systems, failure modes, and so on. Gholizadeh et al. [25] proposed an improved FMEA model to evaluate the risk level of a plane wing by combining the genetic algorithm and fuzzy sets theory. The FMEA method is effective in analysing, identifying, and addressing failure modes that may harm a system’s performance during the design and production stages. By utilizing FMEA, we can enhance a system’s performance. However, conventional FMEA has been criticized by some researchers as it possesses some drawbacks that cannot be ignored [26]. FMEA is a human-made evaluation method which heavily depends on experts’ subjective opinions and experience. Eliminating the subjective and fuzziness of human-made evaluations is a significant challenge that needs to be addressed. In this work, we choose the Dempster–Shafer evidence theory to handle this issue.

The Dempster–Shafer (D-S) evidence theory is a widely used uncertainty reasoning theory [27,28,29,30,31]. It is an effective tool for knowledge reasoning and decision making under uncertain environments, often used to deal with uncertain information in classification [32,33,34], clustering [35,36,37], group decision making [38,39], and so on [40,41,42,43]. N$\tilde{u}$nez et al. [44] introduce a model for implication rules based on the Dempster–Shafer evidence theory and conditional fusion equation, capable of capturing the uncertainty involved in the data and in the knowledge models as well. Liu et al. [45] suggested a method of fusing models based on evidence theory applied to degradation modelling and data analysis. The effectiveness of this model has been demonstrated through various application scenarios.In [46], D-S evidence theory was adopted to the reliability optimization design. Hui et al. [47] used a support vector machines model based on evidence theory to resolve conflicting results from each model, increasing the classification accuracy in multi-bearing fault diagnosis. In [48], D-S evidence theory was adopted to a new approach to validate an engineering heat transfer system model when faced with epistemic uncertainties. The results show the method has advantages over traditional methods. Zhang et al. [49] suggested a new D-S evidence theory-based weighted data fusion approach and tested it on randomly generated datasets and vehicle classification datasets, showing the method to be effective. Lin et al. [50] improved the Dempster combination rule based on the Euclidean distance and applied it to a multi-sensor fault diagnosis modelling of a complex system. Yu et al. [51] proposed a new data fusion method based on event-driven and D-S evidence theory, acquiring high-accuracy fusion results with low control energy overhead. In [52], the authors developed a water quality forecasting model that utilizes a recurrent neural network and an enhanced evidence theory. Zhang et al. [53] proposed an epistemic uncertainty analysis approach based on D-S evidence theory, transforming the standard uncertainty analysis problem into a duo of probabilistic uncertainty analysis problems. An D-S evidence theory-based empirical measure of uncertainty with exponential function form that can overcome some limitations of some previous methods was proposed in [54]. Mao et al. [55] applied an uncertainty algorithm grounded in fuzzy theory, rough set theory, and D-S evidence theory to handle a multi-index uncertainty issue in an electric power system.

D-S evidence theory was also combined with the FMEA method because of its ability to deal with subjective and uncertain assessments. Certa et al. [56] adopted the D-S evidence theory in the failure mode, effects and criticality analysis (FMECA), allowing experts to express interval-valued judgments under an uncertain environment. Wang et al. [57] suggested an advanced FMEA methodology based on Dempster–Shafer evidence theory by integrating an evidential network to enhance the risk evaluation process. The D-S evidence theory was used in the FMECA of a ballast water system onboard a tanker ship in [58]. Measures in the D-S evidence theory can also model the uncertainty in FMEA experts [59]. However, the scores for the same FMEA item from different FMEA experts may be different and conflicting. In this case, it may lead to the deviation of fusion results from reality when different bodies of evidence are combined with conflict scores from different FMEA experts. Therefore, managing potentially conflicting assessments is a problem if we want to apply D-S evidence theory to FMEA. In this paper, we choose the Dempster–Shafer evidence theory to handle experts’ subjective evaluations. Furthermore, the Gaussian model is used to pre-process potentially conflicting evaluations made by experts.

The rest of this paper is organized as follows. In Section 2, the preliminaries are introduced. The proposed FMEA method based on the Gaussian model and D-S evidence theory is introduced in Section 3. In Section 4, the application process and experimental results are analysed and discussed. The conclusion is in Section 5.

## 2. Preliminaries

### 2.1. Dempster–Shafer Evidence Theory

Preliminaries of the Dempster–Shafer evidence theory [60,61] are briefly introduced in this section.

**Definition 1**. *Frame of discernment*

*Let us suppose $\Theta =\{\theta_{l}=1,2,3\ldots L\}$ is a complete mutually exclusive set composed of all possible outcomes which can be recognized. Then the set is called the frame of discernment.*

**Definition 2**. *Basic belief assignment (BBA)*

*The basic belief assignment is a function from the power set of* Θ *to [0,1] which satisfies the following conditions:*

(1) $$m\left(\emptyset \right)=0,\sum_{A\subseteq \Theta }m\left(A\right)=1$$

**Definition 3**. *Dempster combination rule*

*Let us suppose that there are two evidences $E_{1}$ and $E_{2}$ under the frame of discernment *Θ* and $m_{1}$ and $m_{2}$ are their basic belief assignments. Then the Dempster combination rule is defined as the following formula:*

(2) $$m\left(C\right)=\left\{\begin{array}{c} \sum_{A⋂B=C,\forall A,B\subseteq \Theta }\frac{m_{1}\left(A\right)m_{2}\left(B\right)}{1-K}\;A⋂B\neq \emptyset \\ 0\;\;\;\;\;\;\;A⋂B=\emptyset \end{array}\right.$$

*where K is defined as:*

(3) $$K=\sum_{A⋂B=\emptyset ,\forall A,B\subseteq \Theta }m_{1}\left(A\right)m_{2}\left(B\right),K<1.$$

### 2.2. Risk Priority Number in FMEA

The risk priority number (RPN) is an important component of FMEA for risk evaluation and ranking [62,63]. It is the basis for the risk assessment of a system. The value of the RPN is the product of three risk factors, namely, *S* (severity), *O* (occurrence), and *D* (detection). *S* refers to the severity of the failure impact. *O* refers to the frequency of occurrence of the cause of failure and *D* refers to the degree of detection of the cause of failure. The risk level for S,O and *D* is generally divided into 10 from 1–10. The greater the severity, the higher the level. Take the risk factor *O* as an example (Table 1) [64], the higher the level, the more frequent the occurrence.

(4) RPN=S×O×D

### 2.3. Gaussian Distribution

Gaussian distribution [65], also known as normal distribution, is a continuous probability distribution density function that is bell shaped, low at both ends, and high in the middle. Its formula is:

(5) $$f\left(x\right)=\frac{1}{\sqrt{2\pi }\sigma }e^{-\frac{{\left(x-\mu \right)}^{2}}{2\sigma^{2}}}$$

where μ refers to the expected value, σ refers to the standard deviation, and $\sigma^{2}$ refers to the variance.

## 3. Improved FMEA Method Based on the Gaussian Model and Evidence Theory

In this section, we introduce the improved FMEA method based on the Gaussian model and evidence theory. First, we simply the frame of discernment to reduce computations. Furthermore, we construct BBA on the basis of the Gaussian model, introduced below. Then we fuse the BBA function by using the modified Dempster combination rule. Furthermore, we finally obtain the modified mean value risk priority number (MVRPN) to rank the FMEA items. We introduce the Gaussian model and modified Dempster combination rule. After this, we describe the calculation steps in detail.

**Figure 1:**
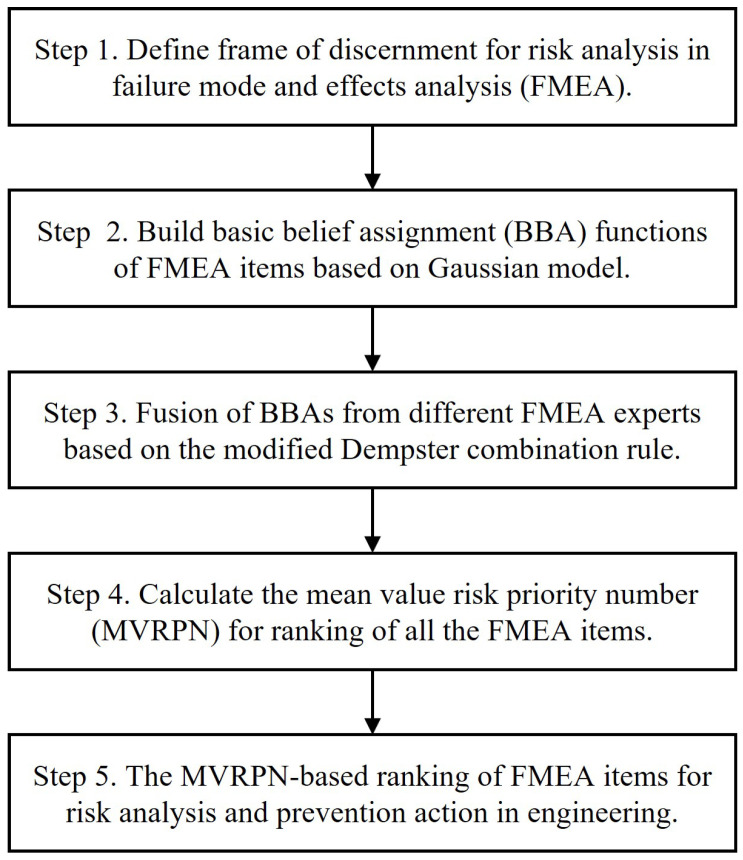
A flow chart of the improved FMEA method based on the Gaussian model and evidence theory.

The detailed calculation steps of the improved FMEA are proposed in Figure 1 and demonstrated as follows.

Step 1 The frame of discernment is defined for risk analysis in failure mode and effects analysis (FMEA).
  The frame of discernment is defined for uncertain information modelling with the basic belief assignment as the first step of applying D-S evidence theory. For FMEA, each risk factor includes 10 levels in the risk assessment and the risk levels are defined in the frame of discernment.
  Suppose that there are *L* experts ($E_{1}$, $E_{2}$…,$E_{l}$) and *N* failure modes in FMEA. Then, the frame of discernment is as follows: $\theta_{i}^{n}=\left\{1,2,3,\ldots ,9,10\right\}$, i=S,O,D, n=1,2,3,4,5…N. We simplified the frame of discernment as: $\theta_{i}^{n}={\left(minX\right|}_{X\subseteq \theta_{i}^{n}}{,minX|}_{X\subseteq \theta_{i}^{n}}+1,\ldots ,maxX{|}_{X\subseteq \theta_{i}^{n}})$, where ${minX|}_{X\subseteq \theta_{i}^{n}}$ and ${maxX|}_{X\subseteq \theta_{i}^{n}}$ refer to the minimum and maximum values of the assessments made by *L* experts on the *i*th risk factors (S,O,D) of the *N*th failure modes, respectively. With the simplified frame of discernment, we can avoid useless calculations.
Step 2 Basic belief assignment (BBA) functions of FMEA items are built based on the Gaussian model.
  It can be seen from the above that the risk level is usually divided into 10 levels ranging from 1 to 10.
  If an expert is greatly influenced by other experts (strong correlation), it means that they are less confident in their own evaluation and willing to follow other evaluations. The higher the degree of correlation, the higher the corresponding degree of generalization. Therefore, by generalizing the evaluation opinions, the opinions of experts cannot be completely opposed. Furthermore, the problem of highly conflicting evidence fusion can be solved.
  Combined with the characteristics of the Gaussian distribution, we can define three kinds of correlation situations, namely, weak, moderate and strong correlation. The corresponding values of the Gaussian distribution are shown in Table 2.
  From Equation (2) we can obtain a mapping *m* from $2^{\theta }$ to [0,1], which satisfies the following condition:
  (6) $$m\left(\emptyset \right)=0,\sum_{X\subseteq \theta_{i}^{n}}m\left(X\right)=1$$
Step 3 Fusion of BBAs from different FMEA experts based on the modified Dempster combination rule.
  The belief degree of each FMEA expert is different in a risk assessment, so the weight of each expert’s assessment in data fusion should be modelled.
  $w_{ij}$ is a weight factor representing the relative weight on the importance of the *j*th expert to the *i*th risk factor ($0\leq w_{ij}\leq 1)$. Based on the classical Dempster combination rule, we multiply the calculated result by $w_{ij}$ to make the fusion result be more reasonable. The new BBA function is recorded as ${\bar{m}}_{ij}^{n}\left(*\right)$.
  (7) $${\bar{m}}_{ij}^{n}\left(C\right)=w_{ij}\times m_{ij}^{n}\left(C\right),C\subseteq \theta_{i}^{n},C\neq \theta_{i}^{n}$$
  (8) $${\bar{m}}_{ij}^{n}\left(\theta_{i}^{n}\right)=1-\sum_{B\subseteq \theta_{i}^{n}}w_{ij}\times m_{ij}^{n}\left(B\right),B\neq \theta_{i}^{n}$$
  where i=O,S,D, n=1,2,3,…,N, *N* refers to the number of FMEA items, and $w_{ij}$ refers to the weight of the *j*th expert for the *i*th risk factor. Using these definitions and modifications on the classical combination rule, we obtain a modified Dempster combination rule for the fusion of BBAs from different FMEA experts:
  (9) $$m_{i,jl}^{n}\left(C\right)=\left(m_{i,j}^{n}⨁m_{i,l}^{n}\right)\left(C\right)=\frac{\sum_{A⋂B=C,A.B\subseteq \theta_{i}^{n}}\left(w_{ij}\times m_{i,j}^{n}\left(A\right)\times w_{il}\times m_{i,l}^{n}\left(B\right)\right)}{1-K}$$
  (10) $$K=\sum_{A⋂B=\emptyset ,A,B\subseteq \theta_{i}^{n}}\left(w_{ij}\times m_{i,j}^{n}\left(A\right)\times w_{il}\times m_{i,l}^{n}\left(B\right)\right)$$
  With Equations (9) and (10), the assessments of two experts can be fused. For all *L* FMEA experts ($E_{1}$, $E_{2}$…,$E_{l}$), the fusion formula is as follows:
  (11) $$m_{i}^{n}=m_{i1}^{n}⨁m_{i2}^{n}⨁m_{i3}^{n}⨁\ldots ⨁m_{iL}^{n}.$$
  In this paper, we default to using equal weights, that is: $w_{ij}$ = 1.
Step 4 The mean value risk priority number (MVRPN) is calculated to rank all the FMEA items.
  After information fusion of FMEA expert assessments, we calculate the RPN. However, the evaluation of each risk factor is represented by a belief function, so we need the mean value of the RPN (MVRPN) to compare the overall risk of each failure mode.
  Assume that the RPN level corresponding to the *i*th failure mode is $RPN_{i}^{1}$, $RPN_{i}^{2}$, …, $RPN_{i}^{m}$ with respect to the belief degrees of different FMEA experts ($P(RPN_{i}^{1})$, …, $P(RPN_{i}^{m})$) defined as:
  (12) $$P\left(RPN_{i}^{1}\right)=m_{i,jl}^{n}$$
  where i=S,O,D, m=1,2,3…N, $RPN_{i}^{m}\subseteq \theta_{i}^{n}$ and the $m_{i,jl}^{n}$ is defined in Equation (9).
  Then,
  (13) $$MVRPN_{i}=\sum RPN_{i}^{m}\times P\left(RPN_{i}^{m}\right).$$
  Therefore, the ultimate RPN according to Equation (4) can be obtained:
  (14) $$RPN=MVRPN_{S}\times MVRPN_{O}\times MVRPN_{D}.$$
Step 5 The FMEA items are ranked based on the MVRPN for risk analysis and prevention action in engineering.
  In practical engineering, such as the air system of an aero turbofan engine, after ranking results of all the FMEA items, the limited resources should be used to take actions to prevent the risk of FMEA items with higher MVRPN values. In this way, the risk level can be decreased to an acceptable level and quality can be guaranteed.

This is the FMEA analysis based on the Gaussian model and evidence theory. In the next section, we apply the proposed method to the FMEA analysis in the gas path of an aviation turbofan engine.

## 4. Application in the Air System of an Aero Turbofan Engine

In this section, we apply the proposed method to the FMEA analysis of the gas path in the air system of an aviation turbofan engine. The experimental results are compared and discussed under different scenarios.

### 4.1. Background of the Aero Turbofan Engine

**Figure 2:**
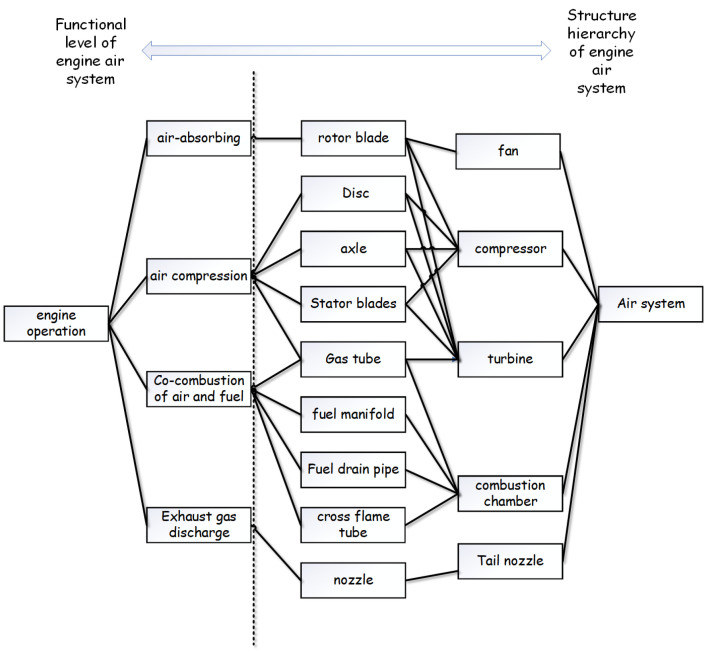
Function–structure level of the engine air system.

The experiment is an application of the proposed FMEA method in the air system of an aero turbofan engine [66,67]. The aero-engine studied in this work is a dual-rotor separate exhaust civil turbofan aero-engine without an afterburner. The aero engine includes fans, low- and high-pressure compressors, a combustion chamber, high- and low-pressure turbine nozzles and some other components. The proposed FMEA method is applied to the air system of an aero turbofan engine. The function–structure level of this engine air system is shown in Figure 2. Then we collect the main failure modes of the components for the air system, as shown in Table 3. After this, the FMEA experts assessments are used to produce a criticality analysis table based on the RPN, shown in the Supplementary Material Table S1: Assessment data for Table 3.

### 4.2. Experiment

The full data used for the experimental analysis in this paper are adopted from [66,67] and shown in the Supplementary Material Tables S1–S3. In [20], the authors took the professional position, job experience, education level and age as evaluation criteria to obtain the weight of each expert. While Mehri Mangeli et al. [68] adopted logarithmic fuzzy preference programming to determine the weight of each FMEA risk factor. In order to more directly observe the difference in results caused by the different expert’s mutual correlation in the Gaussian model, we assume that the weights of the different experts are the same. Taking the risk assessment of FMEA ID 101 as an example, we briefly describe the whole calculation process with the proposed method.

After simplifying the framework of discernment for the FMEA analysis, we build the new BBA function. Assume that three experts are weakly related and the weight of each expert’s assessment is the same. Then, the new BBA functions are calculated as follows:

Expert 1 ($E_{1}$): $m_{O1}^{1}\left(3\right)=0.1,m_{O1}^{1}\left(4\right)=0.8,m_{O1}^{1}\left(5\right)=0.1$

Expert 2 ($E_{2}$): $m_{O2}^{1}\left(2\right)=0.1,m_{O2}^{1}\left(3\right)=0.8,m_{O2}^{1}\left(4\right)=0.1$

Expert 3 ($E_{3}$): $m_{O3}^{1}\left(2\right)=0.1,m_{O3}^{1}\left(3\right)=0.8,m_{O3}^{1}\left(4\right)=0.1$

Then, we first fuse the BBA functions of Expert 1 ($E_{1}$) and Expert 2 ($E_{2}$) (The order has no effect on the final result):

*K* = $\sum_{A⋂B=\emptyset ,A,B\subseteq \theta_{O}^{1}}\left({\bar{m}}_{O,1}^{1}\left(A\right)\times {\bar{m}}_{O,2}^{1}\left(B\right)\right)=0.84$

$m_{O,12}^{1}\left(2\right)=\left({\bar{m}}_{O,1}^{1}\left(A\right)⨁{\bar{m}}_{O,2}^{1}\left(B\right)\right)=\frac{\sum_{A⋂B=2,A.B\subseteq \theta_{O}^{1}}\left(m_{O,1}^{1}\left(A\right)\times m_{O,2}^{1}\left(B\right)\right)}{1-K}=0$

$m_{O,12}^{1}\left(3\right)=\left({\bar{m}}_{O,1}^{1}\left(A\right)⨁{\bar{m}}_{O,2}^{1}\left(B\right)\right)=\frac{\sum_{A⋂B=3,A.B\subseteq \theta_{O}^{1}}\left(m_{O,1}^{1}\left(A\right)\times m_{O,2}^{1}\left(B\right)\right)}{1-K}=0.5$

$m_{O,12}^{1}\left(4\right)=\left({\bar{m}}_{O,1}^{1}\left(A\right)⨁{\bar{m}}_{O,2}^{1}\left(B\right)\right)=\frac{\sum_{A⋂B=4,A.B\subseteq \theta_{O}^{1}}\left(m_{O,1}^{1}\left(A\right)\times m_{O,2}^{1}\left(B\right)\right)}{1-K}=0.5$

$m_{O,12}^{1}\left(5\right)=\left({\bar{m}}_{O,1}^{1}\left(A\right)⨁{\bar{m}}_{O,2}^{1}\left(B\right)\right)=\frac{\sum_{A⋂B=5,A.B\subseteq \theta_{O}^{1}}\left(m_{O,1}^{1}\left(A\right)\times m_{O,2}^{1}\left(B\right)\right)}{1-K}=0$

After this we fuse the BBA function of Expert 3 and the fusion result of the BBA functions from Expert 1 and Expert 2.

*K*=$\sum_{A⋂B=\emptyset ,A,B\subseteq \theta_{O}^{1}}\left({\bar{m}}_{O,12}^{1}\left(A\right)\times {\bar{m}}_{O,3}^{1}\left(B\right)\right)=0.55$

$m_{O,123}^{1}\left(2\right)=\left({\bar{m}}_{O,12}^{1}\left(A\right)⨁{\bar{m}}_{O,3}^{1}\left(B\right)\right)=\frac{\sum_{A⋂B=2,A.B\subseteq \theta_{O}^{1}}\left(m_{O,12}^{1}\left(A\right)\times m_{O,3}^{1}\left(B\right)\right)}{1-K}=0$

$m_{O,123}^{1}\left(3\right)=\left({\bar{m}}_{O,12}^{1}\left(A\right)⨁{\bar{m}}_{O,3}^{1}\left(B\right)\right)=\frac{\sum_{A⋂B=3,A.B\subseteq \theta_{O}^{1}}\left(m_{O,12}^{1}\left(A\right)\times m_{O,3}^{1}\left(B\right)\right)}{1-K}=\frac{8}{9}$

$m_{O,123}^{1}\left(4\right)=\left({\bar{m}}_{O,12}^{1}\left(A\right)⨁{\bar{m}}_{O,3}^{1}\left(B\right)\right)=\frac{\sum_{A⋂B=4,A.B\subseteq \theta_{O}^{1}}\left(m_{O,12}^{1}\left(A\right)\times m_{O,3}^{1}\left(B\right)\right)}{1-K}=\frac{1}{9}$

Then, using Equations (12) and (13), we obtain:

$RPN_{O}^{1}=2,P\left(RPN_{O}^{1}\right)=0$

$RPN_{O}^{2}=3,P\left(RPN_{O}^{2}\right)=\frac{8}{9}$

$RPN_{O}^{3}=4,P\left(RPN_{O}^{3}\right)=\frac{1}{9}$

$MVRPN_{O}=\sum_{m=1}^{3}RPN_{O}^{m}\times P\left(RPN_{O}^{m}\right)=0+3\times \frac{8}{9}+4\times \frac{1}{9}=3.111$

Similarly, we calculate the value of other two risk factors: $MVRPN_{S}=6.899$, $MVRPN_{D}=2$.

According to Equation (14), we obtain the final fused RPN value: RPN=$MVRPN_{S}$×$MVRPN_{O}$×$MVRPN_{D}$ = 42.86.

This is the calculation process of obtaining the RPN value under weak correlation condition through FMEA analysis based on the Gaussian model and evidence theory. Table 4 shows the result of part of the S,O and *D* fusion data calculated with the proposed method (the full data is tabulated in the Supplementary Material Table S2: Full data of Table 4). Among them, the probability level of failure occurrence with FMEA ID No. 803 adopts a moderate correlation for the fusion because the data cannot be fused under weak correlation conditions. However, no significant influence is identified on the overall experimental data analysis. After completing the FMEA analysis under weak correlation conditions, FMEA analysis under medium correlation conditions was continued, as shown in Table 5 (the full data is tabulated in the Supplementary Material Table S3: Full data of Table 5).

### 4.3. Result and Discussion

After calculating the RPN value under weak and moderate correlation conditions, we compare the calculation results with the original data. The original data is obtained by taking the arithmetic average of the RPN values of different experts. Under weak correlation conditions, compared with the RPN value calculated by the average value algorithm, 63.08% of the data difference is 0–5, 29.23% of the data difference is 5–10 and 7.69% of the data difference is above 10. It can be seen that there is a gap between the data obtained under the weak correlation conditions and the data calculated by the average value method. Under moderate correlation conditions, compared with the RPN value calculated by the average value algorithm, 69.23% of the data difference is 0–1, 29.23% of the data difference is 1–5 and 1.54% of the data difference is above 5. It can be seen that the two parts of the data are basically consistent with each other.

**Figure 3:**
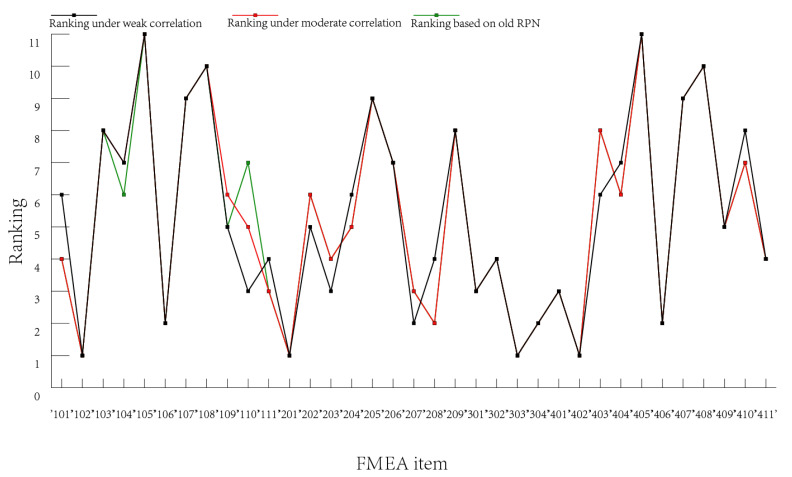
Comparison of the ranking of failure modes.

**Figure 4:**
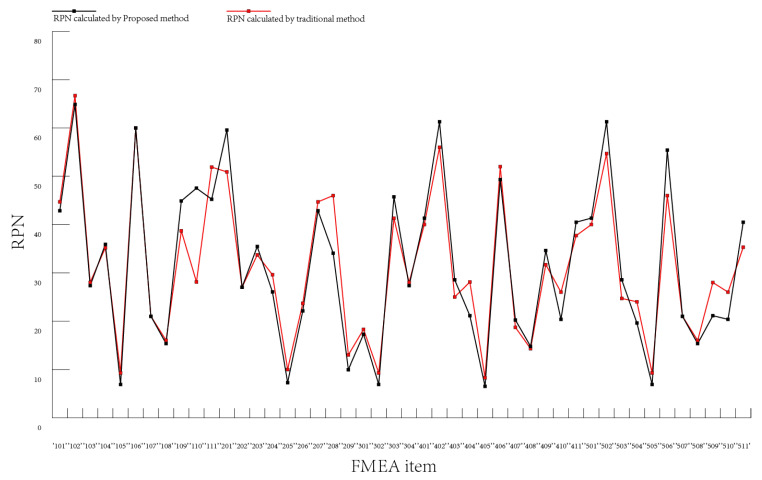
Comparison of the RPN values under weak correlation conditions and the old RPN.

**Figure 5:**
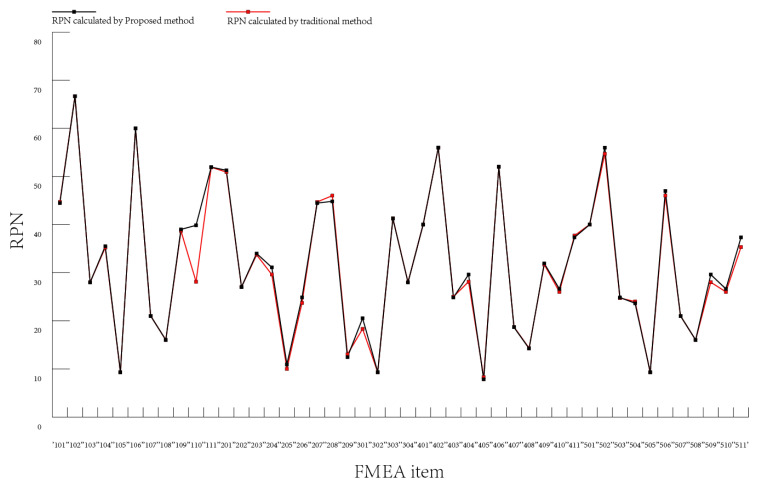
Comparison of the RPN values under moderate correlation conditions and the old RPN.

From the perspective of ranking the failure modes, we can see from Figure 3 that there is little gap between the rank under the weak correlation conditions and the original data. The rank under moderate correlation conditions is consistent with the original data rank. From the perspective of RPN values, Figure 4 and Figure 5 show the comparison of the calculated RPN values with the old RPN values under weak and moderate correlation, respectively. There is a small difference between the new RPN values calculated under the weak correlation conditions and the old RPN values, while the new RPN values calculated under the medium correlation conditions are closely fitted with the old RPN values. These results are consistent with each other in most cases, showing the availability and effectiveness of the proposed method.

There are different assessments from different FMEA experts and the risk level may be conflicting for information fusion. The traditional method is to take the average expert evaluation value as the final evaluation result. The proposed method does not take into account whether the degree of influence between experts is large or small. We only choose a neutral condition. We combine the characteristics of the Gaussian model to determine weak, moderate or strong correlations in FMEA assessments. Under weak correlation conditions, FMEA experts determine the risk assessment value at a certain value more accurately with a small degree of influence among the experts themselves. Therefore, there will be a differences between the RPN value calculated by the average value method and the results calculated by the proposed method. However, the assessment data will be more convincing because the correlation degree among the experts is considered. While under moderate correlation conditions, the degree of correlation is well-situated. There is a smaller gap between the proportion of expert assessment values and the proportion of their peripheral values, explaining that the influence among experts under moderate correlation conditions is higher than under weak correlation conditions. Under this condition, the RPN values are similar to those calculated by the average value method. Above all, FMEA is a subjective assessment method. We use the Gaussian method to model different risk levels from different experts for the fusion of FMEA assessments, especially when there is conflicting assessments on an FMEA item.

## 5. Conclusions

FMEA is a quantitative and qualitative method for risk assessment which is widely used and the RPN is a useful measure of evaluating failure modes in a system. In this paper, an improved FMEA method based on the Gaussian model in the evidence theory framework was proposed for risk analysis of the air system of an aero turbofan engine. Firstly, we simplify the frame of discernment for risk analysis to simplify the calculation of RPN values. Then, based on the Gaussian model, we deal with the problem that the Dempster combination rule cannot handle conflicting evidence of different scores from different experts. Meanwhile, the modified Dempster combination rule was used to fuse FMEA expert assessments. Finally, the RPN values were calculated to rank FMEA items. The Gaussian model effectively describes the degree of mutual influence between experts when making risk assessments. The weak, moderate or strong correlation in the Gaussian model refers to the low, moderate or high degree of mutual influence between experts, respectively. The results obtained under different conditions of risk analysis have different fitting degrees with the original data, consistent with previous research results. The proposed method is flexible in dealing with RPN calculations under different expert correlation conditions. The final experimental results show that the proposed method is convincing for risk analysis in the air system of an aero turbofan engine.

The limitations and possible following work of this study are as follows. (1) The weight of each risk factor is not taken into consideration. (2) The weight of each FMEA expert is assumed to be equal. (3) Other risk factor apart from *O*, *S* and *D* may be needed.

## Figures and Tables

**Table 1:** Ranking level of risk factor *O*.

Frequent the Occurrence	Risk Level
Exceedingly high	10
Definitely high	9
Very high	8
High	7
Moderate high	6
Moderate low	5
Low	4
Very low	3
Definitely low	2
Exceeding ly low	1

**Table 2:** Gaussian distribution parameters in three correlations.

Related Situation	Standard Deviation	Variance	Generalized Scaling
Strong Correlation	σ = 1.2	$\sigma^{2}$ = 1.44	7
Moderate Correlation	σ = 1	$\sigma^{2}$ = 1	5
Weak Correlation	σ = 0.5	$\sigma^{2}$ = 0.25	3

**Table 3:** The main failure modes of the components of the air system.

Component	Failure Modes	Component	Failure Modes
Rotor blade	deformation, flexural, corrosion, rustiness, Scaling, creep, scuff, crack, fracture, wear, slide rail block falling	Turbine nozzle	deformation, flexural, corrosion, rustiness, Scaling, creep, scuff, crack, fracture, wear, slide rail block falling
Disc	crack, fracture, burst, surge, Stall, Flutter, deformation, buckling, over-speed	Diffuser	blow-by, crush, Indentation
Axle	abnormal sound, wear, bending, fracture	Fuel nozzles	Carbon Deposition, corrosion, Ablation, blockage
Stator blades	deformation, flexural, corrosion, rustiness, Scaling, creep, scuff, crack fracture, wear, slide rail block falling	Flame tube	Ablation, crack, deformation burn crack, burned-through, over-temperature, smoking, falling block
		Nozzle	crack, blockage, exhaust temperature overrun

**Table 4:** Calculated data and compared results under weak correlation conditions.

Key Comp.	Failure Mode		*O*	*S*	*D*	New RPN	Old RPN	RPN Difference
	ID	Description	Fused Value	Fused Value	Fused Value	The Weights Are the Same		
Rotor blade	101	deformation	3.111	6.889	2.000	42.863	44.7	−1.837
	102	crack	2.000	8.111	4.000	64.888	66.7	−1.812
	103	fracture	1.000	9.111	3.000	27.333	28.0	−0.667
	104	corrosion	1.889	6.111	3.111	35.912	35.2	0.712
	105	wear	1.111	3.111	2.000	6.913	9.3	−2.387
	106	flexural	2.000	6.000	5.000	60.000	60.0	0.000
	107	slide block fall	1.000	7.000	3.000	21.000	21.0	0.000
	108	scuff	3.000	5.111	1.000	15.333	16.0	−0.667
	109	rustiness	1.889	6.111	3.889	44.893	38.7	6.193
	110	scaling	2.000	6.111	3.889	47.531	28.1	19.431
	111	creep	3.111	6.889	2.111	45.242	51.9	−6.658
Disc	201	crack	1.889	8.111	3.889	59.586	50.9	8.686
	202	fracture	1.000	9.000	3.000	27.000	27.0	0.000
	203	burst	1.889	8.889	2.111	35.446	33.7	1.746
	204	surge	2.889	8.111	1.111	26.034	29.6	−3.566
	205	Stall	3.111	2.111	1.111	7.296	10.0	−2.704
	206	flutter	2.889	6.889	1.111	22.111	23.7	−1.589
	207	deformation	3.111	6.889	2.000	42.863	44.7	−1.837
	208	buckling	1.111	6.000	5.111	34.070	46.0	−11.930
	209	overspeed	3.111	2.889	1.111	9.985	13.0	−3.015
axle	301	abnormal sound	4.000	3.889	1.111	17.283	18.3	−1.017
	302	wear	1.111	3.111	2.000	6.913	9.3	−2.387
	303	bending	1.889	5.889	4.111	45.732	41.3	4.432
	304	fracture	1.000	9.111	3.000	27.333	28.0	−0.667

**Table 5:** Calculated data and compared results under moderate correlation conditions.

Key Comp.	Failure Mode		*O*	*S*	*D*	New RPN	Old RPN	RPN Difference
	ID	Description	Fused Value	Fused Value	Fused Value	The Weights Are the Same		
Rotor blade	101	deformation	3.336	6.664	2.000	44.462	44.7	−0.238
	102	crack	2.000	8.336	4.000	66.688	66.7	−0.012
	103	fracture	1.000	9.321	3.000	27.963	28.0	−0.037
	104	corrosion	1.679	6.336	3.336	35.489	35.2	0.289
	105	wear	1.400	3.336	2.000	9.341	9.3	0.041
	106	flexural	2.000	6.000	5.000	60.000	60.0	0.000
	107	slide block fall	1.000	7.000	3.000	21.000	21.0	0.000
	108	scuff	3.000	5.336	1.000	16.008	16.0	0.008
	109	rustiness	1.679	6.336	3.664	38.978	38.7	0.278
	110	scaling	1.716	6.336	3.664	39.837	28.1	11.737
	111	creep	3.336	6.664	2.336	51.932	51.9	0.032
Disc	201	crack	1.679	8.336	3.664	51.282	50.9	0.382
	202	fracture	1.000	9.000	3.000	27.000	27.0	0.000
	203	burst	1.679	8.664	2.336	33.981	33.7	0.281
	204	surge	2.664	8.336	1.400	31.090	29.6	1.490
	205	Stall	3.336	2.336	1.400	10.910	10.0	0.910
	206	flutter	2.664	6.664	1.400	24.854	23.7	1.154
	207	deformation	3.336	6.664	2.000	44.462	44.7	−0.238
	208	buckling	1.400	6.000	5.336	44.822	46.0	−1.178
	209	overspeed	3.336	2.664	1.400	12.442	13.0	−0.558
axle	301	abnormal sound	4.000	3.664	1.400	20.518	18.3	2.218
	302	wear	1.400	3.336	2.000	9.341	9.3	0.041
	303	bending	1.679	5.664	4.336	41.235	41.3	−0.065
	304	fracture	1.000	9.321	3.000	27.963	28.0	−0.037

## Data Availability

All relevant data are within the paper.

## Supplementary Materials

The following supporting information can be downloaded at: https://www.mdpi.com/article/10.3390/e25050757/s1, Table S1: Assessment data for Table 3. Table S2: Full data of Table 4. Table S3: Full data of Table 5.
